# “Time wasted by health professionals is time not invested in patients”: time management practice and associated factors among health professionals at public hospitals in Bahir Dar, Ethiopia: a multicenter mixed method study

**DOI:** 10.3389/fpubh.2023.1159275

**Published:** 2023-07-21

**Authors:** Berihun Alemayehu Addis, Yared Mulu Gelaw, Fantu Abebe Eyowas, Tewodros Worku Bogale, Zewdu Bishaw Aynalem, Habtamu Alganeh Guadie

**Affiliations:** ^1^Department of Midwifery, Bahir Dar Health Science College, Bahir Dar, Ethiopia; ^2^School of Public Health, College of Medicine and Health Sciences, Bahir Dar University, Bahir Dar, Ethiopia; ^3^Health Systems Strengthening (HWIP), Jhpiego-Ethiopia, Bahir Dar, Ethiopia; ^4^Department of Midwifery, College of Medicine and Health Sciences, Injibara University, Injibara, Ethiopia; ^5^Department of Nursing, College of Medicine and Health Sciences, Injibara University, Injibara, Ethiopia

**Keywords:** time management, factors, health professionals, public hospitals, Bahir Dar

## Abstract

**Background:**

Time management is of utmost importance in healthcare facilities since time squandered by health professionals is time not invested in patients, and this affects the quality of care given to patients. This study aimed to assess time management practice and factors affecting it among health professionals at public hospitals in Bahir Dar, Ethiopia.

**Methods:**

Institution-based cross-sectional study supplemented with a qualitative study was conducted from April 21 to May 20, 2022, among health professionals working in public hospitals in Bahir Dar, Ethiopia. A simple random sampling technique was used to select 416 study participants. A pretested self-administered questionnaire was used to collect quantitative data, and an interviewer guide was used to collect qualitative data to complement quantitative data. Purposive sampling was used in the qualitative study, and 12 in-depth interviews were carried out till saturation was reached. The quantitative data were entered into Epi Data version 4.0 and analyzed using SPSS 25.0 whereas the qualitative data were analyzed manually using thematic analysis. To identify the associated factors, bi-variable and multivariable logistic regression analyses were used. The degree of associations was interpreted using odds ratio and 95% confidence interval at <0.05 *p*-value.

**Results:**

Of 416 invited participants, 410 of them participated in the study yielding a 98.5% response rate. The magnitude of time management practice among health professionals was 66.1% (95% CI: 61.5–70%). Age ranges 25–29 (AOR = 3.961, 95% CI: 1.068, 14.682) and 30–34 (AOR = 6.240, 95% CI: 1.640, 23.749), planning (AOR = 6.032, 95% CI: 3.478, 10.463), compensation and benefits packages (AOR = 1.888, 95% CI: 1.077, 3.309), responsible to work (AOR = 2.119, 95% CI: 1.192, 3.768), time waster (AOR = 1.855, 95% CI: 1.058, 3.251) and staff shortage (AOR = 0.535, 95% CI: 0.319, 0.896) were factors associated with time management practice. From the qualitative study, two major themes and five categories have emerged.

**Conclusion and recommendations:**

Healthcare facilities could improve their time management practices by providing training on planning, being a low time-waster and highly responsible at work, and designing compensation and benefits packages.

## Background

Hospitals are crucial 24/7 healthcare service delivery environments that demand healthcare staff with effective and efficient management skills. Time management is one of these skills (1). With the advent of the industrial revolution, this skill has evolved into the concept of completing tasks efficiently and effectively (2, 3). It is the practice of arranging, organizing, scheduling, planning, and budgeting how much time must be spent on specific tasks (4, 5). It is the ability to say no, limit interruptions, make a good investment in time, and control time carefully (6).

Time management is how time is planned, controlled, and utilized and is essential in healthcare organizations (7, 8). Effective time management is vital for providing high-quality healthcare because patients wait for less, colleagues and other health workers feel less job-related stress, and activities are completed quickly without interfering with the work of others (9). Time management is life management (10). It is a key to achieving the lifestyle balance one desire (11). It boosts productivity, reduces burnout, encourages development, and improves personal and professional satisfaction (9, 12, 13). Time management has an impact on productivity and performance. It could assist employees in working more efficiently, meeting deadlines, producing higher-quality work, prioritizing all job duties, achieving their goals more quickly, taking new possibilities, and successfully building a company (14).

Managing time effectively can benefit both the personal life and the organization. It improves the ability to complete tasks, make smarter decisions, and achieve complete control over essential priorities (15). Poor time management, on the other hand, has been linked to poor job quality, low productivity, a negative impact on the career path, and high levels of stress (5). Similarly, health professionals with poor time management skills frequently work overtime to complete their tasks. As a result, they have less personal and family time, causing more stress. It can also make them more exhausted, cause burnout, and have a negative influence on their health (16). Furthermore, an employee who is unable to manage his or her time has the potential to make conflict in the office. Missed deadlines, financial losses, stressful relationships, and job loss are all potential consequences of poor time-management skills (17).

Ultimately, the efficiency and success of any organization, whether private or public, are largely determined by its employees’ effective use of limited resources, time (18). It is one of the most valuable assets for any organization (19) and is uniformly distributed to everyone, regardless of age, position, or working in the public or private sector (18). Time is an important resource that continues to pass away from us without going back, hence, tasks must be performed with time (3). The more time saved the better and continuous improvement occurs. Time management could be viewed as one of the most important competitive tools for improving organizational performance (20) and it was indicated that effective time management is a remedy for organizational effectiveness (21). Time management practices, however, are contextual and could be influenced by the organization’s culture and leadership style (22). Similarly, prioritization of tasks and activities, delegation, good meeting management, and planning for professional and personal concerns are all factors influencing time management practice (6).

Although time management is a main health professional discipline that reflects the quality of taking responsibility and accountability (23, 24), nearly a quarter (24.6%) of health professionals claimed two regular latecomers out of their five coworkers (25). Similarly, evidence showed that health professionals spent up to 37% of their time on administrative activities and working at their desks. Only 9 to 22% of their time is spent with patients in hospital practice, and more than half of their time is spent on activities unrelated to patients (26). Non-clinical work significantly squanders health professionals’ time and reduces their performance efficiency. One study found that physicians 44.64% and nurses 11.34% of their time spent outside patient rooms (27). To the worst, a growing body of literature describes most health professionals spending more time on non-productive tasks such as gossiping, answering phone calls, and socializing among themselves than on their actual responsibilities (8). Lengthy paper-based documentation was also believed to be the main time management barrier in hospitals (28). This trend is concerning since investing less time in patients affects healthcare workers’ satisfaction as well as patient education and health promotion activities, which can lead to incorrect prescriptions and treatment errors (29).

Many nations including health professionals, particularly in developing countries like Africa, have a lack of time management culture, which can be damaging to both the organization and the worker (30). The majority of people believe they have too much to do and not enough time, and they attribute their unmet goals, low productivity, and poor performance to a lack of time (31). Every organization has implemented time management practices, yet there is still a gap in achieving productivity and increased employee performance (32). In this regard, a number of studies have been conducted worldwide and in some parts of Ethiopia to assess time management practice and its contributing factors among health professionals. But, the majority of such studies used a cross-sectional study design which did not explore participants’ time management experiences. Again, no similar study has been conducted in the study area. To fill this gap, this study used a cross-sectional study supplemented with a qualitative study among healthcare professionals. Furthermore, the results of this study would also help hospitals, policymakers, patients, and future researchers as an input by providing information on time management practice and the factors influencing it and to take remedial actions. Therefore, this study tried to assess time management practice and associated factors among health professionals working in public hospitals in Bahir Dar City.

## Methods

### Study design and setting

An institution-based cross-sectional study supplemented with a qualitative study was conducted at Public Hospitals in Bahir Dar City from April 21 to May 20, 2022. Bahir Dar is the capital city of Amhara National Regional State which is located in the Northwest part of Ethiopia and is 565 km far away from the capital city of Ethiopia, Addis Ababa. This city has three public hospitals: Felege Hiwot Referral Hospital, Tibebe Ghion specialized hospital, and Addis Alem primary hospital. About 871, 707, and 158 altogether 1,736 health professionals were assigned to different working departments of these hospitals, respectively.

### Study populations

All health professionals who are working in the public hospitals of Bahir Dar City for at least 6 months were the source population for the study. But, health professionals who were on maternity or annual leave or those who are seriously ill during the data collection period were excluded.

### Sample size and sampling

The sample size for the quantitative part was calculated based on the single population proportion formula by considering the following assumptions: the percentage of time management practice as 56.4% (32), a 95% level of confidence, a 5% margin of error, and a non-response rate of 10%. By adding a 10% nonresponse rate, the final sample size was 416. For the qualitative component of the study, 12 participants who were not involved in the quantitative study were interviewed until saturation is reached.

**Figure 1:**
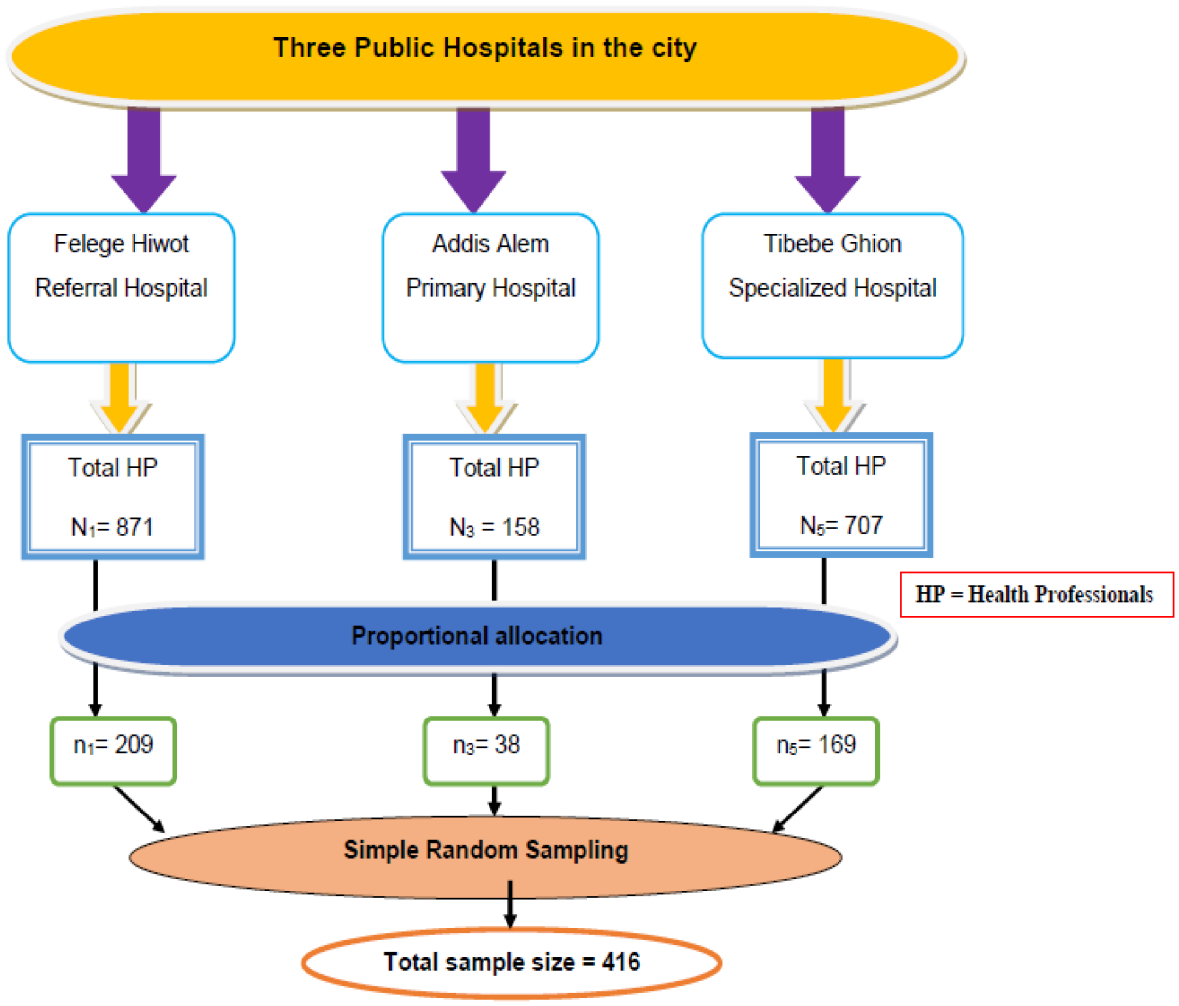
Schematic presentation of sampling procedure on factors associated with time management practice among health professionals working at public hospitals in Bahir Dar city, Northwest Ethiopia, 2022.

To recruit participants for this study, proportional allocation with simple random sampling was used (Figure 1). For qualitative data, a purposive sampling technique was employed to select 12 study participants.

### Study variables

#### Data collection tool and measurement

Data collection tools were developed from previous related works of literatures (8, 32, 33) (Table 1). The tool was initially developed in English then translated to Amharic and then pretested to ensure its cultural acceptance and understanding by the study participants and data collectors. For the quantitative part, data were collected using a structured, anonymous, and self-administered questionnaire. The Cronbach alpha test was used to check the reliability of the tool using the data obtained from the pre-test. The overall tool’s value was 0.83, indicating that the instrument is reliable. The content validity of the instrument was reviewed by two public health professionals. To collect data, participants were given questionnaires to fill out and data collectors instructed them on how to do so while also describing the study’s aims. To avoid low response rates, health professionals from all shifts were included. Completed questionnaires were collected on the subsequent day. For the qualitative part, data were collected by the principal investigator using field notes. A semi-structured interview guide was used to conduct in-depth interviews to explore the experience of time management.

**Table 1:** Dependent and independent study variables.

Dependent variable	Time management practice
Independent variables	
Socio-demographic variables	Age, sex, marital status, educational status, residence, type of profession, work experience and average monthly salary
Personal variables	Procrastination, time-wasters and punctuality
Organizational and administrative variables	Organizational policy and strategy, performance appraisal, work environment, compensation and benefit, recognition and promotion, workload and staff shortage
Employee performance	Planning, implementation, and responsibility.

#### Time management practice

To measure health professionals’ time management practice, five questions were asked. Each item had a five-point Likert scale: 0 representing strongly disagree, 1 indicating disagree, 2 denoting neutral, 3 denoting agree, and 4 representing strongly agree with a minimum score of 0 and maximum 20. Healthcare professionals with total scores of 13 or more (≥ 65%) were categorized as having good time management practices, while those with total scores below 13 (< 65%) were classified as having poor time management practices (32).

### Procrastination

Assessed using four items each of which was scored on a 5-point Likert scale. It was considered as high if the responses were ≥ the mean score value and low if the responses were below the mean score value (32, 34).

#### Time wasters

Categorized as high if the responses were ≥ the mean score value and low if the responses were below the mean score value (32).

#### Organization policy and strategy

Assessed using three items, each of which was valued on a five-point Likert scale. If the response was above or equal to the mean score value, it was classified as satisfied otherwise as unsatisfied (32).

#### Performance appraisal

Measured using three items, each of which was assessed on a five-point Likert scale. If the responses were above or equal to the mean score value, it was classified as satisfied, and if they were below the mean score value, it was classified as unsatisfied (32).

#### Work environment

The quality of the working environment both its physical qualities and the extent to which it supplied meaningful work conditions. It was measured using a 5-point Likert scale for each of the five items. If the responses were above and equal to the mean score value, it was considered as a good work environment, and if not a bad work environment (32).

#### Compensation and benefit

Measured using three items, each of which was calculated on a five-point Likert scale. If the responses were ≥ the mean score value, it was categorized as satisfied, and if they were below the mean score value, it was categorized as unsatisfied (32).

#### Recognition and promotion

Measured using four items, each of which was rated on a five-point Likert scale. If the responses were above or equal to the mean score value, it was categorized as satisfied and below as unsatisfied (32).

#### Responsibility

Assessed using 3 items, each of which was rated on a five-point Likert scale. If the responses were ≥ the mean score value, it was classified as high, and if they were below the mean score value, it was classified as low (32).

#### Workload

Respondents who work >39 h per week were considered as having a “workload” otherwise “no workload” (35).

#### Shortage of staff

Self-reported insufficient number of health professionals in the health facilities where they work (8).

### Data management and analysis

The responses of the study participants were coded and entered into Epi-info 7.2 and then exported to Statistical Package for Social Science (SPSS) version 25 for analysis. Descriptive statistics and summary measures of the variables were conducted to see the characteristics of the study participants. To determine the association between the outcome and the independent variables, crude and adjusted odds ratios (OR) with a 95% confidence interval were carried out using bi-variable and multi-variable logistic regression analysis. Variables from the bi-variable logistic regression that had a *p*-value of <0.2 were further entered into the multivariable logistic regression. Model fitness was checked by using a Hosmer-Lemeshow goodness-of-fit test (*p* = 0.386). Finally, *p*-value < 0.05 was considered statistically significant for all explanatory variables at multivariable logistic regression. Qualitative data were analyzed using manual thematic analysis.

### Data quality assurance

A pre-test was conducted using 5% of the sample among health professionals in Injibara General Hospital to assess instrument simplicity, flow, and consistency. Some modifications like specific language and typographic corrections were made based on the results obtained from the pre-test. Data was collected by one-day trained three data collectors and two supervisors. Data completeness and consistency were checked by the supervisor and investigators.

## Results

### Socio-demographic characteristics of the respondents

**Figure 2:**
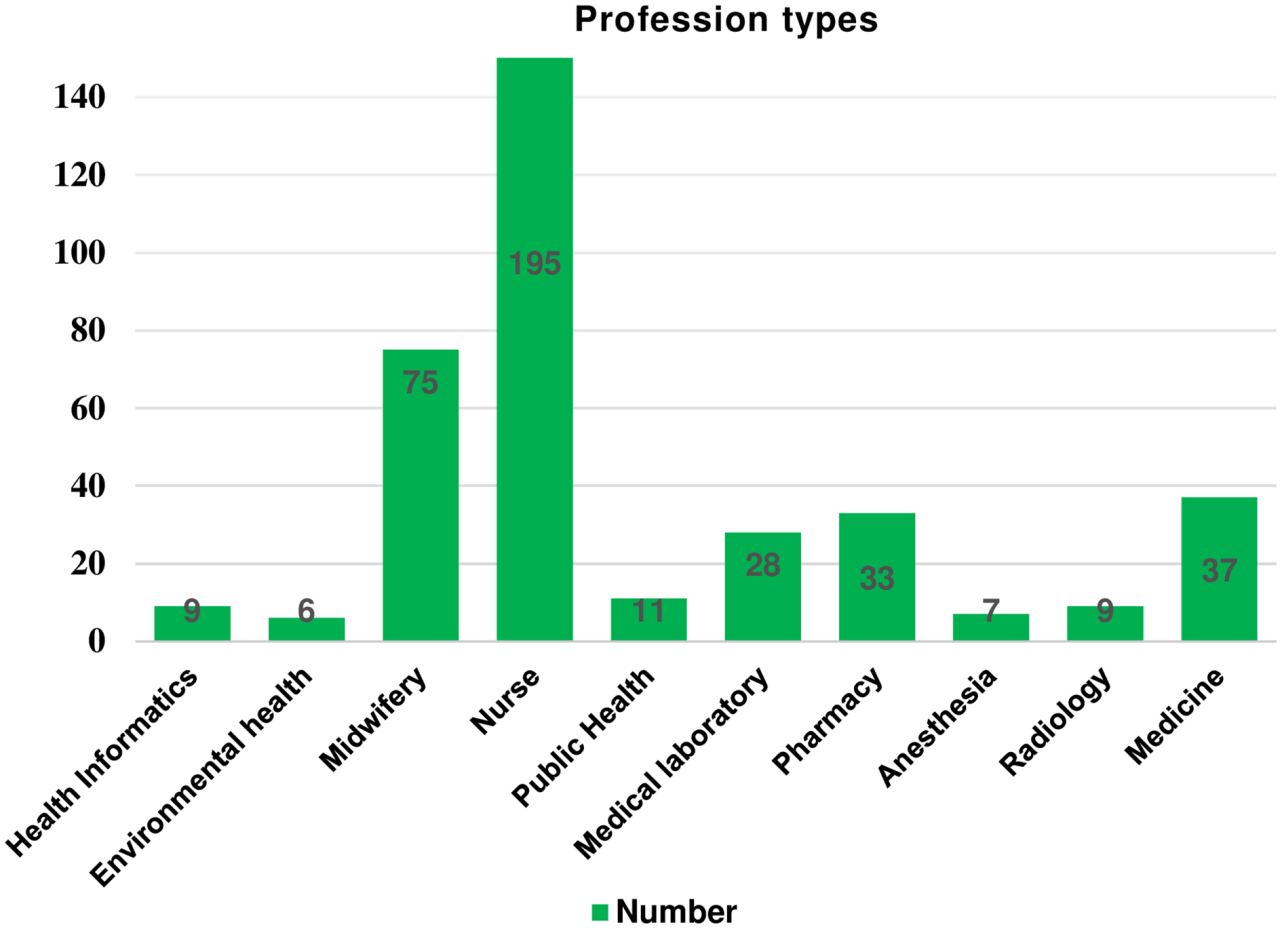
Professional types of study participants working at public hospitals in Bahir Dar city, Northwest Ethiopia, 2022, (*n* = 410).

In this study, 410 study participants were involved, with a 98.5% response rate. Of these, 242 (59.0%) were males. The age range of the respondents was 20 to 58 years with a mean of 31.64 (SD ± 0.285). The majority of study participants were married, BSc holders, and rural background residents 287 (70.0%), 336 (82.0%), and 238 (58.0%) respectively. The mean (SD) monthly salary of the respondent was 7837.88 (±179.441) ETB. Based on quartile salary classification: 109 (26.6%) participants had a monthly salary of 7,086–9,000 Birr ($133.7–169.8) (Table 2). The majority of the study participants were nurses (195; 47.6%) (Figure 2).

**Table 2:** Socio-demographic characteristics of health professionals working at public hospitals in Bahir Dar city, Northwest Ethiopia, 2022, (*n* = 410).

Variables	Category	Numbers	Percent
Age (in years)	20–24	18	4.4
	25–29	154	37.6
	30–34	132	32.2
	≥35	106	25.9
Sex	Male	242	59.0
	Female	168	41.0
Marital status	Single	114	27.8
	Married	287	70.0
	Divorced	9	2.2
Educational status	Diploma	26	6.3
	BSc	336	82.0
	MSc and above	48	11.7
Address of the respondent	Urban background	172	42.0
	Rural background	238	58.0
Work experience (in years)	≤5	183	44.6
	6–10	150	36.6
	>10	77	18.8
Monthly salary (ETB)	<6500.00	100	24.4
	6500.00–7085.00	104	25.4
	7086.00–9000.00	109	26.6
	>9000.00	97	23.7

### Organizational and administrative-related factors

**Figure 3:**
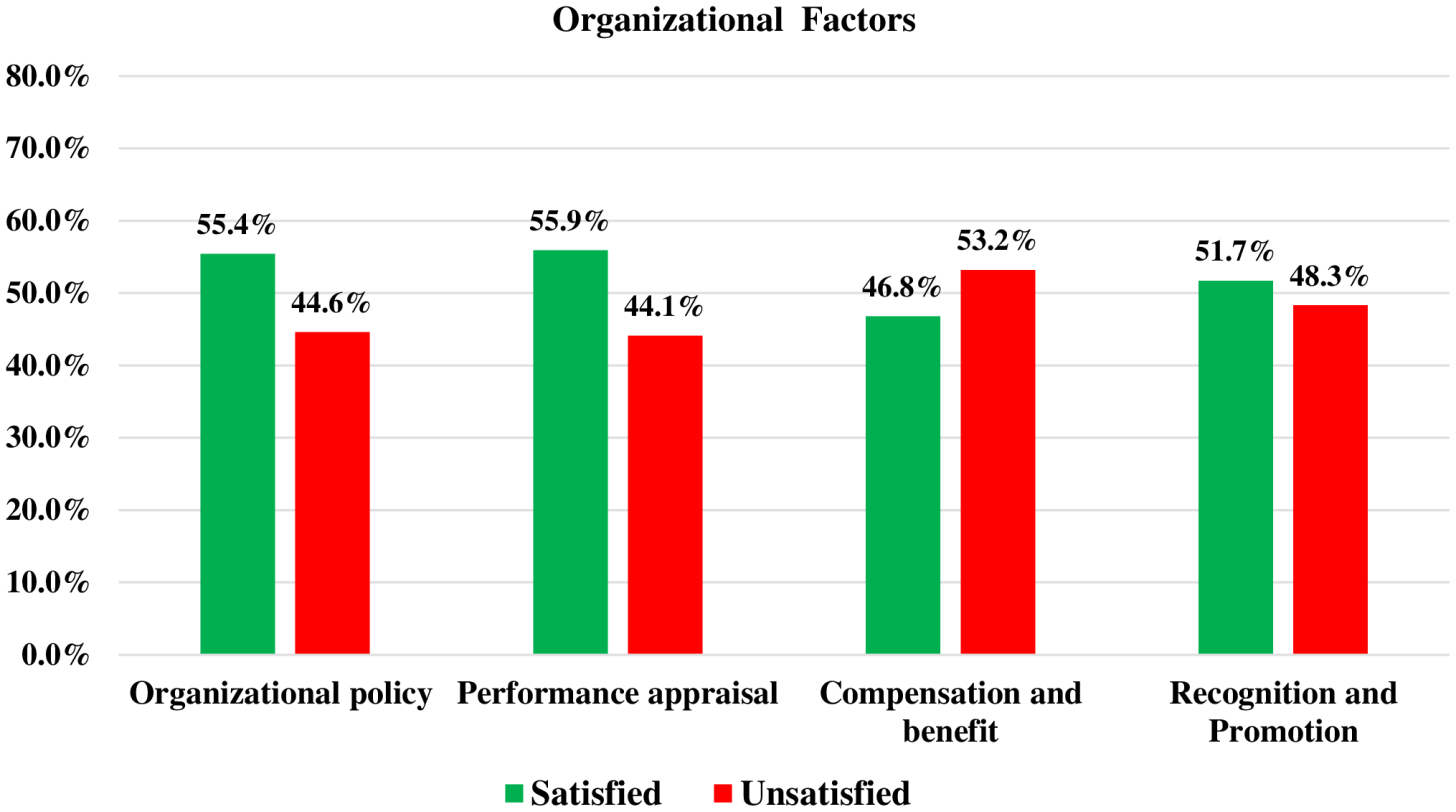
Organizational and administrative related factors among health professionals working at public hospitals in Bahir Dar city, Northwest Ethiopia, 2022, (*n* = 410).

Only 195 (47.6%) study participants reported that the work environment in the hospital was good, 252 (61.5%) of them reported that there is an insufficient number of health professionals in the health facilities where they work, 158 (38.5%) of them described there is workload. Additionally, 183 (44.6%) of them were dissatisfied with the organization’s policy and 218 (53.2%) of the participants were unsatisfied with the hospital’s compensation and benefits systems, respectively (Figure 3).

### Personal and employee performance-related factors

Though the majority of respondents, 233 (56.8%), were punctual for their work, 227 (55.4%) of them had high procrastination. Furthermore, only 236 (57.6%) of them were highly responsible for their work (Table 3).

**Table 3:** Personal and employee performance-related factors among health professionals working at public hospitals in Bahir Dar City, Northwest Ethiopia, 2022, (*n* = 410).

Variables	Category	Numbers	Percent
Punctuality	Yes	233	56.8
	No	177	43.2
Time waster	High	192	46.8
	Low	218	53.2
Procrastination	High	227	55.4
	Low	183	44.6
Planning	Yes	222	54.1
	No	188	45.9
Implementation	High	230	56.1
	Low	180	43.9
Responsibility	High	236	57.6
	Low	174	42.4

### Time management practice among health professionals

In this study, the magnitude of health professionals who had good time management practice was found to be 271 (66.1%) 95% CI: 61.5–70%. The mean (SD) score for time management practice was 17.00 (±3.651). About 170 (41.5%) health professionals agree that they have both short and long-term goals. Additionally, 181 (44.1%) of them agree that they execute tasks and give delegation when necessary (Table 4).

**Table 4:** Health professionals’ agreement level on time management practice at public hospitals in Bahir Dar City, Northwest Ethiopia, 2022, (*n* = 410).

No	Activities	SD	D	*N*	A	SA
		*N*	%	*N*	%	*N*	%	*N*	%	*N*	%
	Having short and long-term goals	38	9.3	58	14.1	100	24.4	170	41.5	44	10.7
	Having a list of priority tasks	19	4.6	67	16.3	119	29.0	184	44.9	21	5.1
	Prioritizing tasks based on importance	13	3.2	48	11.7	97	23.7	187	45.6	65	15.9
	Having a schedule for each activity	26	6.3	66	16.1	105	25.6	175	42.7	38	9.3
	Executing tasks and giving delegation when appropriate	26	6.3	50	12.2	93	22.7	181	44.1	60	14.6

Key: SD, strongly disagreed; D, disagreed; N, neutral; A, agree; SA, strongly disagreed.

### Factors affecting health professionals’ time management practice

From various variables that were entered into the bi-variable model, the following were significantly associated with good time management practice at a *p*-value <0.2: sex, age, educational level, background address, work experience, organizational policy and strategy, performance appraisal, compensation and benefit, recognition and promotion, workload, staff shortage, punctuality, time-waster, procrastination, planning, implementation, and responsibility. Then, these variables were further entered into a multivariable logistic regression model using the backward LR method to control confounders. Age, compensation and benefit, shortage of staff, time-waster, planning, and responsibility remained statistically significant with good time management practice at a *p*-value <0.05.

Age was found to be significantly associated with time management practice. Health professionals in the 25–29 and 30–34 age ranges were nearly four times (AOR = 3.961, 95% CI: 1.068, 14.682) and six times (AOR = 6.240, 95% CI: 1.640, 23.749) more likely to practice good time management at work than those with age groups of 20–24 years, respectively. The practice of time management was shown to be significantly associated with compensation and benefits packages in the hospital. Health workers who were satisfied with the hospital compensation and benefits packages were 89% higher (AOR = 1.888, 95% CI: 1.077, 3.309) to have good time management practices than unsatisfied workers. Similarly, health professionals who were effective at planning their tasks were about 6 times (AOR = 6.032, 95% CI: 3.478, 10.463) more likely to have good time management practices than those who failed to plan.

When compared to low responsible employees for their work, highly responsible health professionals were 2.12 times (AOR = 2.119, 95% CI: 1.192, 3.768) more likely to have good time management practices. Additionally, it was shown that health professionals who wasted less time were 86% (AOR = 1.855, 95% CI: 1.058, 3.251) more likely to have good time management practices than those who wasted more time. Furthermore, health professionals who reported a staff shortage were 46.5% (AOR = 0.535, 95% CI: 0.319, 0.896) less likely to have good time management practices than those who said there was an adequate number of healthcare professionals at the hospital (Table 5).

**Table 5:** Binary logistic regression analyses of socio-demographic, organizational, personal, and employee performance-related factors to time management practice among health professionals working at public hospitals in Bahir Dar City, Northwest Ethiopia, 2022, (*n* = 410).

Variables	Categories	Time management		COR (95%CI)	AOR (95%CI)
		Good *N* (%)	Poor *N* (%)		
Sex	Male	153 (37.3%)	89 (21.7%)	1	1
	Female	118 (28.8%)	50 (12.2%)	1.373 (0.901,2.092)	1.224 (0.715, 2.096)
Age (year)	20–24	6 (1.5%)	12 (2.9%)	1	1
	25–29	100 (24.4%)	54 (13.2%)	3.704 (1.316,10.420)	3.961 (1.068, 14.682)^*^
	30–34	98 (23.9%)	34 (8.3%)	5.765 (2.008,16.552)	6.240 (1.640, 23.749)^*^
	≥35	67 (16.3%)	39 (9.5%)	3.436 (1.195,9.883)	3.333 (0.872, 12.732)
Marital status	Single	74 (18.0%)	40 (9.8%)	1	
	Married	192 (46.8%)	95 (23.2%)	1.092 (0.692,1.724)	
	Divorced	5 (1.2%)	4 (1.0%)	0.676 (0.172,2.659)	
Level of education	Diploma	9 (2.2%)	17 (4.1%)	1	1
	BSc holder	227 (55.4%)	109 (26.6%)	3.934 (1.699,9.109)	1.670 (0.546, 5.113)
	MSc & above	35 (8.5%)	13 (3.2%)	5.085 (1.818,14.225)	1.314 (0.327, 5.277)
Background address	Urban	121 (29.5%)	51 (12.4%)	1.392 (0.915,2.118)	1.545 (0.909, 2.626)
	Rural	150 (36.6%)	88 (21.5%)	1	1
Experience (in years)	≤5	103 (25.1%)	80 (19.5%)	1	1
	6–10	116 (28.3%)	34 (8.3%)	2.650 (1.638,4.287)	1.850 (0.914, 3.744)
	>10	52 (12.7%)	25 (6.1%)	1.616 (0.923,2.826)	1.179 (0.478, 2.909)
Average monthly salary (ETB)	<6,500	60 (14.6%)	40 (9.8%)	1	
	6,500–7,085	76 (18.5%)	28 (6.8%)	1.810 (1.003,3.264)	
	7,086–9,000	72 (17.6%)	37 (9.0%)	1.297 (0.739,2.279)	
	>9,000	63 (15.4%)	34 (8.3%)	1.235 (0.693,2.202)	
Organizational policy	Satisfied	171 (41.7%)	56 (13.7%)	2.534 (1.667, 3.854)	1.274 (0.707, 2.297)
	Unsatisfied	100 (24.4%)	83 (20.2%)	1	1
Performance appraisal	Satisfied	173 (42.2%)	56 (13.7%)	2.616 (1.719, 3.982)	1.660 (0.967, 2.851)
	Unsatisfied	98 (23.9%)	83 (20.2%)	1	1
Work environment	Good	133 (32.4%)	62 (15.1%)	1.197 (0.794, 1.805)	
	Bad	138 (33.7%)	77 (18.8%)	1	
Compensation and benefit	Satisfied	136 (33.2%)	56 (13.7%)	1.493 (0.987, 2.259)	1.888 (1.077, 3.309)^*^
	Unsatisfied	135 (32.9%)	83 (20.2%)	1	1
Recognition & Promotion	Satisfied	155 (37.8%)	57 (13.9%)	1.922 (1.269, 2.911)	1.147 (0.605, 2.177)
	Unsatisfied	116 (28.3%)	82 (20.0%)	1	1
Workload	Present	180 (43.9%)	102 (24.9%)	1	1
	Absent	91 (22.2%)	37 (9.0%)	1.394 (0.886, 2.192)	1.104 (0.571, 2.134)
Shortage of staff	Yes	96 (23.4%)	62 (15.1%)	0.681 (0.449, 1.034)	0.535 (0.319,0.896)^*^
	No	175 (42.7%)	77 (18.8%)	1	
Punctuality	Yes	177 (43.2%)	56 (13.7%)	2.791 (1.831, 4.253)	1.111 (0.606, 2.036)
	No	94 (22.9%)	83 (20.2%)	1	1
Time waster	High	94 (22.9%)	98 (23.9%)	1	1
	Low	177 (43.2%)	41 (10.0%)	4.501 (2.893, 7.003)	1.855 (1.058, 3.251)^*^
Procrastination	High	136 (33.2%)	91(22.2%)	1	1
	Low	135 (32.9%)	48 (11.7%)	1.882 (1.233, 2.873)	0.623 (0.339, 1.142)
Planning	Yes	194 (47.3%)	28 (6.8%)	9.988 (6.110, 16.327)	6.032 (3.478, 10.463)^**^
	No	77 (18.8%)	111 (27.1%)	1	
Implementation	High	184 (44.9%)	46 (11.2%)	4.276 (2.766, 6.611)	1.003 (0.530, 1.899)
	Low	87 (21.2%)	93 (22.7%)	1	
Responsibility	High	188 (45.9%)	48 (11.7%)	4.294 (2.780,6.632)	2.119 (1.192, 3.768)^*^
	Low	83 (20.2%)	91 (22.2%)	1	1

Where: 1 = Reference, ^**^p< 0.001, ^*^p< 0.05.

### Results of the qualitative study

Twelve in-depth interviews were carried out among health professionals. We stopped the in-depth interviews with 12 participants because of idea saturation. The participants were between the ages of 29 and 45 years. Eight (67%) of the participants were men. The majority of them (83.3%) were married. Two participants had a diploma, four (33%) had a BSc, and six (50%) had MSc in educational level. Likewise, 7 (58.3%) of them had worked for more than ten years. Three of them were nurses, two were midwives, two were public health officers, three were medical doctors, and the rest two were pharmacists. The findings from the qualitative study identified two major themes and five sub-themes that were associated with health professionals’ time management practice. The themes included organizational and personal levels. The organizational level theme was divided into three subthemes: management system, lack of orientation training, and workload. Technology and planning were the two sub-themes that made up the personal level theme.

### Management system

Participants in the in-depth interviews highlighted that one of the main factors that could affect health professionals’ time management practice was the organization management system. For example, one interviewee, (40, F) expressed her view

“M*any of us have trouble in managing time, regardless of our profession type. But, I think, a poor organizational management system was the top reason for employees' time waste. …organizations with this system require an excessive number of meetings to address rumors and gossip, have ineffective communication among staff, lack staff encouragement systems either weekly or monthly and create low staff morale. These all may affect the status of time management practice of employees.”*

### Lack of induction or orientation training

Some interviewees noted that a barrier to health professionals’ time management practice was the lack of induction training for new staff. Participants claim that the hospital human resource office largely focuses on recruitment, selecting, and placing new applicants, but they pay less attention to this pressing issue. For example, one participant said:

*“I've been working here for three years, yet throughout that time I've never seen a welcoming new employee system. To your surprise, new employee is not immediately made familiar with the work environment, their coworkers, and the institution's policies. As a result, a new employee can arrive late and leave early to the work area”* (interview with a 29-year-old male participant, P2).

### Workload

Health professionals’ practice to manage their time was considerably affected by the workload, which was mostly brought by a staffing shortage. Interviewees stated that the presence of workload forces employees to work under pressure. If health professionals are under stress, patient care at the hospital will be compromised and both the facility and the patients will incur higher resource wastage including time. For example, a 33-year-old, male interviewee expressed his view by saying.

*“Hmm…all activities in the hospital such as history taking, physical examination, documentation, and counseling requires time. In particular, I wrote all patient paperwork by hand, which is time-intensive. Although I am good at being punctual, I have to do these activities and counsel a lot of patients. For example, after getting all care and receiving an appointment card, a patient says wait doctor I have one more question. This affects my time and also deters other patients from being treated on time”* (interview with a 33-year-old male participant, P3).

### Technology

Participants emphasized that although technology makes life easier, it may also have a detrimental impact on time. Employees may become distracted by technology, which causes time to be wasted and results in poor attention to work. For instance: a 36-year-old female participant, P4 stated that:

*“…personally, I do not think there is a health professional who does not pay too much time and attention to social media currently. For example, there is my friend who frequently uses a phone for the internet connection, plays telephone games, and follow-up a series of movies. With the advent of technology, staffs are prone to open social media sites like Facebook, Imo, LinkedIn, Telegram, Twitter, and YouTube, which results in an improper use of their work time.”*

### Planning

According to those who participated in the interviews, employees’ planning abilities were the main barrier to time management practice. For example, one participant said.

*“Because, by efficiently managing my time, I could avoid becoming overwhelmed and I can balance my tasks. I think my future will be determined not by tomorrow but by what I do now. Time is like a river, in my opinion. One cannot touch the same water twice since the flow that has passed will not ever occur.”* (Interview with a 40-year-old female participant, P1). Similarly, a 37-year-old male participant, P6 said that:

*“… proper time management could be practiced through developing certain key skills such as planning, scheduling and delegating daily tasks.”*

Also, another participant described:

*“….Effective time management requires regular practice, not just one morning. Though I was poor in time management, I improved it over time by prioritizing daily tasks. Time management is a skill that can be learned.*” (Interview with a 45-year-old, male participant, P5).

## Discussion

In healthcare facilities, time management is critical since time wasted by healthcare workers is time not spent with patients, and this has a detrimental impact on the quality of treatment provided to patients (9, 36). Therefore, this study was aimed at assessing time management and factors affecting it among health professionals at public hospitals in Bahir Dar, Ethiopia.

In this study, the magnitude of good time management practice among health professionals was 66.1% (95% CI: 61.5–70%). This finding is comparable with the study done in Hebron hospital in Palestine where 69.5% of health workers had good time management (37). However, this finding is lower when compared to a study conducted in Egypt where 79.9% of health professionals had practiced good time management (38). The disparity could be attributed to differences in socio-demographic characteristics, sample sizes, and study settings.

This finding was higher as compared to studies conducted in Ethiopia (32, 39), Nigeria (30), UAE (37), and Saudi Arabia (40) where time management practice was 56.4, 33, 51, 49 and 46% among health professional, respectively. The discrepancy could be due to differences in the study setting, study time, and sample sizes. In addition, some previous studies had a narrower emphasis, such as only on nursing professionals (37, 38) unlike this study. Furthermore, the possible reason for this difference may be the study population. In this study, the participants were from specialized, referral, and primary hospitals, whereas in North Gondar, Ethiopia study (32) the participants were solely from primary hospitals. The reason could be due to the fact that health professionals working at high levels of health institutions were more experienced, giving value to time and aged since age was the most predictor variable in this study.

In this study, age was found to be significantly associated with time management practice. Health professionals in the 25–29 and 30–34 age ranges were 3.96 times and 6.24 times more likely to practice good time management at work than those with age groups of 20–24 years, respectively. This finding is supported by results from Bangladesh (41) and a meta-analysis study done in Canada (13). The explanation might be that as health professionals’ age increases, they may have gained useful expertise that will aid them in managing their time. *“…Effective time management requires regular practice, not just one morning. Though I was poor in time management, I improved it over time. …Time management is a skill that can be learned and taught.*” (Interview with a 45-year-old, male participant, P5).

The practice of time management was shown to be significantly associated with compensation and benefits packages in the hospital. Health workers who were satisfied with the hospital compensation and incentives were 89% higher (AOR = 1.888, 95% CI: 1.077, 3.309) to have good time management practices than unsatisfied workers. This finding is consistent with research done in Ethiopia (32). This may be because hospitals that offer compensation and incentives packages will retain and encourage health professionals, create a healthier sense of competitiveness among employees, and enable staff to stick with their workplace to get the designed incentives. These all, therefore, result in good time management practices among health professionals.

In this study, health professionals who reported a staff shortage were 0.535 times (AOR = 0.535, 95% CI: 0.319, 0.896) less likely to have good time management practices than those who said there were an adequate number of healthcare professionals at the hospital. This finding is in agreement with a study done at Mankweng Hospital, South Africa in which 47% of respondents strongly agreed that a staffing deficit resulted in poor time management, which ultimately results in poor patient care (8). The likely reason might be that a lack of adequate staff forces employees to work under pressure. If health professionals are under stress, patient care at the hospital will be compromised and both the facility and the patients will incur higher resource wastage including time.

In addition, health professionals who were effective at planning their tasks were about 6 times more likely to have good time management practices than those who failed to plan. This finding is in harmony with studies conducted in Ethiopia (32), the UAE, and Pakistan on time management and academic success (37, 42). Interview with a 37-year-old male participant, P6 said that *“… proper time management could be practiced through developing certain key skills such as planning, scheduling and delegating daily tasks.”* A possible reason could be that when health professionals set clearly defined goals through planning, it helps them stay focused on their objectives and acts as a roadmap for them. Planning also helps them to prioritize the activities that need to be completed, by handling the urgent and important tasks first. These all attributed to good time management practice among health professionals. A study by Rimmer Abi lends credence to this idea. A good time manager is someone who takes a few time in the morning to prioritize daily tasks and estimate the amount of time needed to complete each one (43).

When compared to low responsible employees for their work, highly responsible health professionals were 2.12 times more likely to have good time management practices. This finding is consistent with a study done in Ethiopia that 56.8% of health professionals took high responsibility for their work (32). Furthermore, it was shown that health professionals who wasted less time on non-productive activities were 86% (AOR = 1.855, 95% CI: 1.058, 3.251) more likely to have good time management practice than those who wasted more time. The most likely explanation is that if health professionals know common time-wasters like postponing tasks without a solid reason, wasting time on social media, miscommunication, holding numerous meetings without an agenda, and reading mailbox and message notifications, they can easily avoid them. Instead, by identifying important and urgent tasks, they could better able to manage their time. This finding is supported by a study done in America given that an institution may improve its service delivery by identifying and eliminating time-wasting activities in the workplace (44). Pitre Cory (45) also found that both time wasters and hijacked time continued to affect the physicians ability to manage their time effectively.

The limitations of this study include using a self-administered questionnaire could result in bias, but adding qualitative data may minimize such possibility. Also, despite conducting a thorough literature search on time management practice among health professionals, no adequate publications were found. As a result, the literature is inconsistent.

## Conclusion

In this study, the magnitude of time management practice among health professionals was found to be at modest magnitude as compared to studies conducted in other parts of Ethiopia. Increased age, satisfaction with compensation and benefits packages, presence of adequate staff number, planning tasks, being low time-waster and highly responsible to work were significant factors to time management practice among health professionals. Healthcare facilities could improve their time management practice by providing training on planning, offering orientation training for their newly recruited staff, being a low time-waster and highly responsible at work, and designing compensation and benefits packages.

## Data availability statement

The original contributions presented in the study are included in the article/supplementary material, further inquiries can be directed to the corresponding author.

## Ethics statement

All methods were performed in accordance with relevant guidelines and regulations. The ethical review board of Bahir Dar University, College of Medicine and Health Science approved the study (approval # medi/15930/2.4.). After explaining purpose of the study, informed consent was obtained from all participants. Again, study participants were informed about their full right to refuse, stop, skip or ignore any questions. Confidentiality was maintained by using anonymous questionnaires. The in-depth interview was performed at a suitable and secure place for participants. The patients/participants provided their written informed consent to participate in this study.

## Author contributions

BA conceived the idea. BA, TB, and ZA contributed to data collection, organized the interviews, and conducted the qualitative thematic analysis. BA, YG, HG, and FE actively participated in methodology, data analysis and interpretation of results. ZA and BA wrote the first draft of the manuscript. All authors contributed to the article and approved the submitted version.

## Conflict of interest

The authors declare that the research was conducted in the absence of any commercial or financial relationships that could be construed as a potential conflict of interest.

## Publisher’s note

All claims expressed in this article are solely those of the authors and do not necessarily represent those of their affiliated organizations, or those of the publisher, the editors and the reviewers. Any product that may be evaluated in this article, or claim that may be made by its manufacturer, is not guaranteed or endorsed by the publisher.

## Abbreviations

AOR: Adjusted odds ratio
BSc: Bachelor of Science
COR: Crude odds ratio
CI: Confidence interval
ETB: Ethiopian birr
MPH: Master of Public Health
MSc: Master of Science
PhD: Doctor of Philosophy
SD: Standard deviation
SPSS: Statistical Package for Social Science
UAE: United Arab Emirates
UK: United Kingdom
USD: United states dollar
